# Suitability and Acceptability of Decentralised Care for People Living with HIV

**DOI:** 10.1007/s10461-026-05144-z

**Published:** 2026-04-29

**Authors:** Brian Dixon, Samuel Zen Kai Yap, Minyoung Baek, Nicola Boyle, Dorarca Lynch, Colm Bergin, Catherine Fleming, Liam Townsend

**Affiliations:** 1 School of Medicine, University of Galway, Galway, Ireland; 2 School of Medicine, Trinity College Dublin, Dublin, Ireland; 3 Department of Infectious Diseases, University Hospital Galway, Galway, Ireland; 4 Department of Infectious Diseases, St James’s Hospital, Dublin, D08 NHY1 Ireland; 5 Department of Clinical Medicine, Trinity College Dublin, Dublin, Ireland

**Keywords:** Model of care, HIV, Community, Outpatient care

## Abstract

HIV management is evolving, with improved long-term survival and patient health. However, the model of care for people living with HIV (PLWHIV) in Ireland has remained unchanged, with care focused in tertiary sites in urban centres. We explored the acceptability of decentralised HIV care at local hospitals, community healthcare settings, or with primary care providers (PCP), and the acceptability of receiving antiretroviral therapy (ART) locally. HIV attendees at two tertiary referral centres were invited to complete a questionnaire recording demographics, transit time to clinic, engagement with PCP, satisfaction with current model of care and acceptability of alternative models on a Likert scale and provide written qualitative feedback. The relationship between acceptance of new models of care and sociodemographic factors were investigated. *N* = 511 (71% male) PLWHIV at two tertiary HIV centres participated. The median time spent traveling to and from clinic was 1 h 50 min. While the current model of care was convenient to the majority, decentralised care models were also acceptable to most participants. Longer travel time was associated with increased acceptance of care locally (β=-0.015, *p* < 0.001), while male sex was associated with acceptance of community-based care (β=-1.11, *p* = 0.007). Care in the community and receipt of ART locally was more acceptable to younger patients. Concerns regarding stigma and lack of local expertise were cited as barriers to changing the current care model. We identify a cohort of PLWHIV for whom an alternative model of care is preferable, and supports the development of a hybrid HIV care model in Ireland.

**Supplementary Information:**

The online version contains supplementary material available at https://doi.org/10.1007/s10461-026-05144-z.

## Introduction

Human Immunodeficiency Virus (HIV) care has evolved significantly over the last thirty years, with the condition transformed from a life-limiting infection to a chronic illness [1]. Lifespan for people living with HIV (PLWHIV) is similar to HIV-negative populations [2]. Therapeutic advances have been remarkable, with multiple once-daily single tablet regimens available, as well as long-acting injectable therapy [3]. There have also been significant changes in the epidemiology of PLWHIV in Ireland, with an increase in migration of people with known HIV diagnoses and a reduction in intravenous drug use as a risk factor acquisition, as well as a growing ageing population [4, 5].

Despite the advances in HIV care and the changing demographics of PLWHIV, the model of HIV care delivery in Ireland has remained unchanged since its implementation in 1988. HIV care began in a single tertiary centre, and while there are now seven tertiary centres providing HIV care, there is no decentralisation of care. Four of these are located in the largest city, Dublin, with the remaining three in the cities of Cork, Galway and Limerick. Antiretroviral therapy (ART) is dispensed directly by hospital pharmacies at these centres. Care is provided by both infectious diseases and genitourinary medicine (sexual health) specialists, with no formal provision of care to PLWHIV by primary care providers or community pharmacists. Additionally, in many cases PLWHIV cannot access their HIV care at their nearest hospital, necessitating them to travel to tertiary centres. This centralised model is in contrast to the model of care for other chronic conditions in Ireland [6]. Time taken to travel to healthcare facilities is well recognised as a barrier to accessing care across multiple conditions, including in resource-rich settings [7–9]. This results in inequitable access to care, which is associated with poor outcomes for chronic diseases [10]. Decentralised care for PLWHIV has demonstrated improved retention in care and cost-effectiveness in low and middle-income countries [11, 12]. However, there are relatively limited data on acceptability and effectiveness of similar models in resource-rich settings [13].

We aimed to explore satisfaction with the current model of care and acceptability of alternative models, including utilising local hospitals, primary care providers and local pharmacies, in PLWHIV attending for care at two tertiary centres in Ireland. We subsequently investigated social and demographic factors associated with acceptance of new models of care, with a view to informing future HIV care models in Ireland and ensuring that HIV remains patient-centred.

## Methods

### Study Setting and Participants

This was a multicentre prospective study at two hospital sites in Ireland: St James’s Hospital (SJH), Dublin, and University Hospital Galway (UHG). There are just over 7,000 PLWHIV attending care in Ireland. Of these, approximately 3,500 PLWHIV attend SJH for care, with approximately 1,200 attending UHG. The study took place between May – August 2025. HIV care, including ART, is available free of charge to all PLWHIV in Ireland. Primary care is free for approximately 42% of the population, based on financial means and comorbid conditions, with the remaining 58% paying for their primary care [14]. Approximately 10% of the Irish population are not registered with any primary care provider. There is no HIV disease registry in Ireland, with individual sites only submitting data relevant to the UNAIDS 95-95-95 goals to the national Health Protection Surveillance Centre. Therefore, there are no data available on the proportion of PLWHIV in Ireland who are registered with a PCP.

### Data Collection and Variables

Patients attending for HIV care at both sites were approached by a member of the study team and provided with a study patient information leaflet. They were then invited to complete an anonymous paper-based survey and questionnaire. Sociodemographic data collected included gender, age (banded into < 24 years of age, 25–49, 50–65, > 65), region of birth (Ireland, Sub-Saharan Africa, South America, Eastern Europe, Central Europe, Western Europe, South and Southeast Asia, and Other), and employment status. Banding of age and region of birth was performed to ensure data would remain non-identifiable. Data collected relating to their HIV care and overall health status included mode of transport used to get to the clinic, time taken to get to and from clinic, year of HIV diagnosis, knowledge of most recent HIV viral load, whether they have a primary care provider, and whether they have to pay for their primary care. Participants were also asked if they have shared their HIV status with their partner, family members, friends, dentist and primary care provider. Participants were not asked to provide the absolute value of their most recent viral load, their most recent CD4 count, or any disease-related outcomes other than duration since diagnosis. Due to the anonymous nature of the data collected, this data could not be retrieved from patient healthcare records after primary data collection.

Satisfaction with the current model of care was assessed using a Likert scale from 1 to 10, where 1 is strongly disagree and 10 is strongly agree. Participants were asked to rate the convenience of their current care, as well as satisfaction with receiving care at their nearest hospital, a community healthcare setting, from their own primary care provider (PCP) or a PCP of their choosing. They were also asked to rate their acceptance of receiving ART from their local pharmacy. Following data collection, these responses were also differentiated into “disagree” (Likert scores 1–3) and “agree” (Likert scores 8–10). Participants were invited to provide additional unrestricted written feedback on HIV care. A copy of the questionnaire used is in Appendix 1.

### Statistical Analysis

Descriptive statistics are reported as medians with interquartile ranges (IQR). Between-site differences were assessed using Chi-square, ANOVA and Wilcoxon ranked-sum tests, as appropriate. Likert scale responses were assessed using a Spearman correlation with a subsequent multivariable linear regression model used to evaluate significant variables found on univariate analysis. A similar approach was taken to evaluating “agree” and “disagree” outcomes, with a multivariable logistic regression model used to evaluate significant univariate variables. Qualitative written feedback was analysed thematically using Braun and Clarke methods to identify common themes. All analysis was performed using Stata version 18.0 (Stata Statistical Software).

## Results

### Participant Characteristics

*N* = 1,600 PLWHIV attended the study sites for HIV care during the study period. Responses were obtained from *n* = 511 (*n* = 379 at SJH, *n* = 132 at UHG), representing 32% of the eligible population during the study period and 11% (511/4,700) of the total PLWHIV cohort at these sites and approximately 7% (511/7,000) of all PLWHIV attending care in Ireland. The majority of respondents were male (*n* = 363, 71%), aged 25–49 (68%), and employed (74%). Most respondents were also registered with a PCP (*n* = 400, 78%). Most patients (56%) were not taking medication other than ART, while approximately two-thirds of patients were aware of their most recent HIV viral load. The median total travel time was one hour and fifty-five minutes. 10% of respondents spent more than five hours travelling to and from clinic. Respondents had been living with HIV for a median of 13 years (IQR 7–18), and *n* = 436 (85%) had shared their status with at least one person. There were some between-site differences, with a higher proportion of males at SJH (76% vs. 58%, Χ^2^ = 16.02, *p* < 0.001) but fewer patients at SJH having a PCP (75% vs. 88%, Χ^2^ = 8.36, *p* = 0.004). The demographics of the overall cohort and by site are shown in Table 1.

**Table 1:** Participant demographics

	Total (*n* = 511)	SJH (*n* = 379)	UHG (*n* = 132)	
Sex, male, *n* (%)	363 (71)	287 (76)	76 (58)	Χ^2^ = 16.02, *p* < 0.001
Age, years; n (%)				
15–24	6 (1)	5 (1)	1 (< 1)	r^2^= 0.001, *p* = 0.83
25–49	345 (68)	257 (68)	88 (67)	
50–65	123 (24)	87 (23)	36 (27)	
> 65	37 (7)	29 (8)	8 (6)	
Region of origin; n (%)				
Ireland	177 (35)	134 (35)	43 (33)	r^2^= 0.002, *p* = 0.38
Sub-Saharan Africa	133 (26)	82 (22)	51 (39)	
South America	106 (21)	91 (24)	15 (11)	
Eastern Europe	26 (5)	20 (5)	6 (5)	
Western Europe	21 (4)	16 (4)	5 (4)	
Central Europe	7 (1)	4 (1)	3 (2)	
South/SE Asia	15 (3)	12 (3)	3 (2)	
Other	26 (5)	20 (5)	6 (5)	
Time with HIV, years; median (IQR)	13 (7–18)	13 (7–18)	12 (6–17)	z = 1.05, *p* = 0.29
Time to travel, minutes; median (IQR)	110 (60–180)	100 (60–180)	110 (60–200)	z= -0.57, *p* = 0.57
Employment; n (%)				
Employed	380 (74)	278 (74)	102 (77)	r^2^= 0.003, *p* = 0.24
Unemployed	68 (13)	50 (13)	18 (14)	
Student/retired	63 (12)	51 (13)	12 (9)	
Taking other medication, yes; n (%)	224 (44)	160 (42)	64 (48)	Χ^2^ = 1.08, *p* = 0.30
Know viral load, yes; n (%)	346 (68)	251 (66)	95 (72)	r^2^= 0.002, *p* = 0.28
Have a PCP, yes; n (%)	400 (78)	284 (75)	116 (88)	Χ^2^ = 8.36, *p* = 0.004
Pay for PCP, yes; n (%)	260 (65)	197 (69)	63 (54)	Χ^2^ = 4.58, *p* = 0.21
Has shared status, yes; n (%)	436 (85)	312 (82)	124 (94)	Χ^2^ = 3.51, *p* = 0.06

Demographics of total cohort and by site

*SJH* St James’s Hospital, *UHG* University Hospital Galway, *PCP* primary care provider

### Model of Care Delivery

**Fig. 1:**
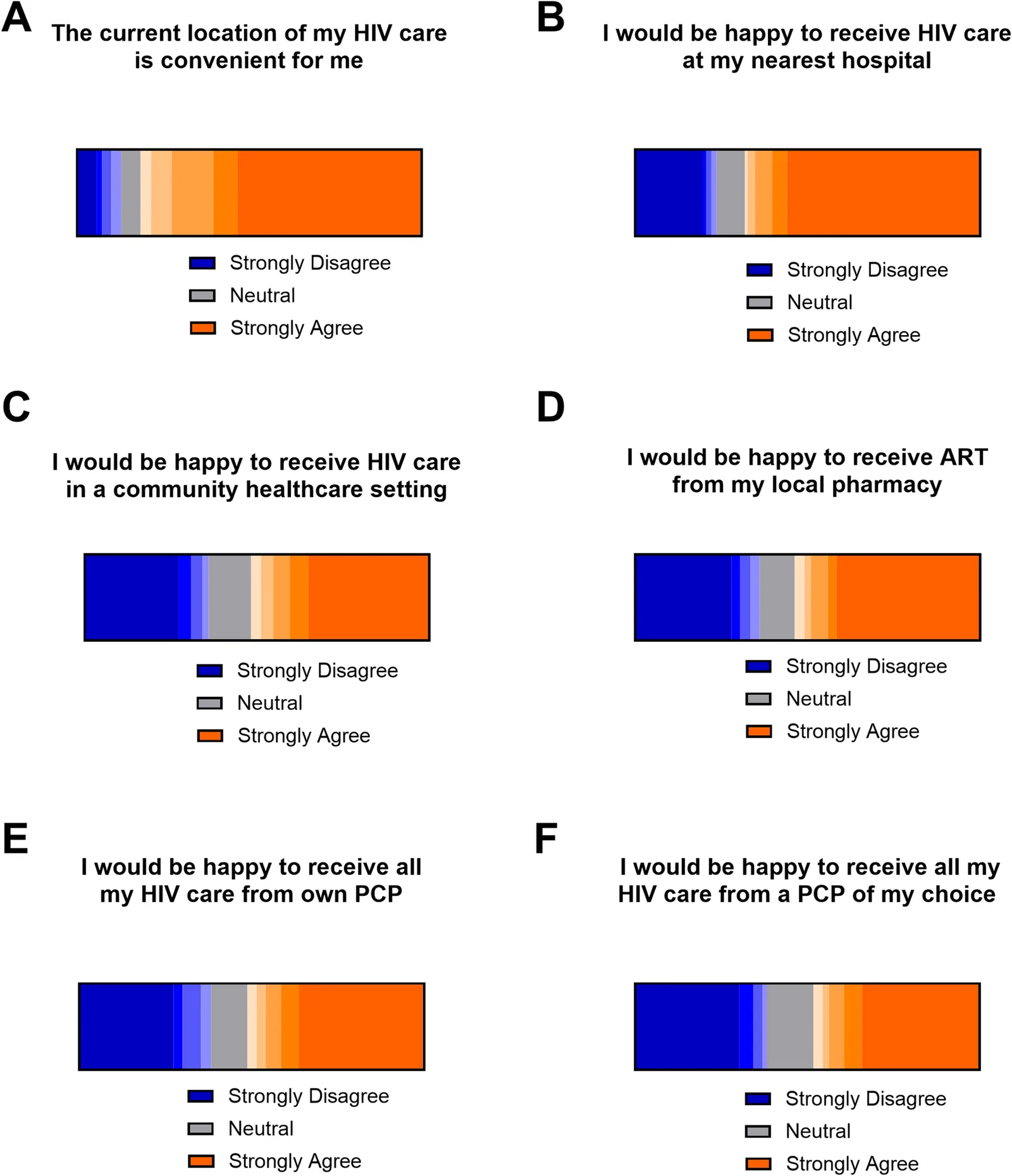
Attitudes towards models of care. Spectrum responses on a Likert scale, ranging from 1 (strongly disagree) to 10 (strongly agree). Domains covered were convenience of current care location (**A**), happiness to receive care at their nearest hospital (**B**). happiness to receive care in a community healthcare setting (**C**), happiness to receive ART from their local pharmacy (**D**). happiness to receive all HIV care from their own PCP (**E**), and happiness to receive all HIV care from a PCP of their choice (**F**)

Under the current model of care, only *n* = 201 (39%) participants were receiving care in their nearest hospital. This was significantly lower in SJH (14%) compared to UHG (55%) (χ^2^ = 21.93, *p* < 0.001). Despite this, the majority of respondents felt that the current location of their care was convenient (Fig. 1A). There were high levels of acceptability towards other potential models of care, with more than half of respondents agreeing that HIV care at their nearest hospital, community healthcare setting or primary care provider would be acceptable (Fig. 1B–E). The majority were also accepting of accessing ART at their local pharmacy (Fig. 1F). There were between-site differences in acceptability of new care models, with attendees at SJH less agreeable to attend their nearest hospital but more accepting of receiving ART from their nearest pharmacy (Supplemental Table 1).

Patient factors associated with acceptability of care models were interrogated. Convenience of current location of HIV care was associated with shorter travel time (r^2^=-0.43, *p* < 0.001). Conversely, a preference for care to be delivered at the nearest hospital to the respondent was associated with longer travel time (r^2^=-0.25, *p* < 0.001). Receiving care at the participant’s nearest hospital was also associated with a more recent HIV diagnosis (r^2^=-0.14, *p* = 0.003). Males were more accepting of receiving care in the community (r^2^ = 0.04, *p* = 0.04), as were older patients (r^2^ = 0.04, *p* = 0.01). Younger age was also associated with receiving care from a PCP of the patient’s own choosing (r^2^ = 0.05, *p* = 0.01), but not with receiving care from the patient’s current PCP. Respondents who were agreeable to receive medication from their local pharmacy were more likely to be born in Ireland, be older, and have a primary care provider. There were no associations seen with having disclosed their HIV status to others or employment status with any of the models of care. Complete results are shown in Table 2. Associations with dichotomised outcomes of “agree” (response scores of 8–10) and “disagree” (response scores of 1–3) demonstrated similar associations to the continuous outcomes (Supplemental Table 2).

These associations were investigated in more detail with multivariable regression models, with all significant variables on univariate analysis included in each model. Following adjustment, acceptability of receiving HIV care at the nearest hospital remained significantly associated with total travel time for both continuous (β coefficient − 0.015, 95% CI -0.02 – -0.01, *p* < 0.001) and dichotomous classification (z=-2.65, *p* < 0.001). Receipt of care in a community healthcare setting was associated with younger age and male sex under both continuous and dichotomous classification. Receipt of ART was not associated with any recorded factors when assessed using a Likert scale. However, increased acceptability of ART from local pharmacies was associated with both younger age and being born in Ireland when dichotomised. These results are shown in full in Table 3 and Supplemental Table 3.

**Table 2:** Acceptance of models of care by patient factor

	Current model is convenient		Care at nearest hospital				ART from local pharmacy		Care from own PCP		Care from PCP of choice	
Sex	r^2^ = 0.01	p = 0.64	r^2^ = 0.04	p = 0.05	r^2^ = 0.04	p = 0.04	r^2^ = 0.03	p = 0.18	r^2^ = 0.01	p = 0.85	r^2^ = 0.01	p = 0.88
Age	r^2^ = 0.03	p = 0.09	r^2^ = 0.03	p = 0.06	r^2^ = 0.04	p = 0.01	r^2^ = 0.05	p = 0.005	r^2^ = 0.02	p = 0.33	r^2^ = 0.05	p = 0.01
Born in Ireland	r^2^ = 0.02	p = 0.24	r^2^ = 0.02	p = 0.24	r^2^ = 0.01	p = 0.90	r^2^ = 0.04	p = 0.04	r^2^ = 0.01	p = 0.73	r^2^ = 0.02	p = 0.53
Time of travel	r^2^ = -0.43	p < 0.001	r^2^ = -0.25	p < 0.001	r^2^ = -0.02	p = 0.61	r^2^ = -0.01	p = 0.78	r^2^ = 0.04	p = 0.40	r^2^ = 0.06	p = 0.18
Time living with HIV	r^2^ = -0.08	p = 0.10	r^2^ = -0.14	p = 0.003	r^2^ = -0.11	p = 0.03	r^2^ = -0.13	p = 0.01	r^2^ = -0.02	p = 0.70	r^2^ = 0.01	p = 0.76
Employed	r^2^ = 0.03	p = 0.19	r^2^ = 0.03	p = 0.09	r^2^ = 0.02	p = 0.58	r^2^ = 0.02	p = 0.40	r^2^ = 0.04	p = 0.06	r^2^ = 0.02	p = 0.39
Has PCP	Χ^2^ = 13.82	p = 0.15	Χ^2^ = 11.03	p = 0.27	Χ^2^ = 12.45	p = 0.19	Χ^2^ = 26.46	p = 0.002	Χ^2^ = 7.04	p = 0.63	Χ^2^ = 4.06	p = 0.91
Disclosed status	Χ^2^ = 2.40	p = 0.98	Χ^2^ = 7.26	p = 0.61	Χ^2^ = 4.20	p = 0.90	Χ^2^ = 5.69	p = 0.77	Χ^2^ = 8.83	p = 0.96	Χ^2^ = 12.22	p = 0.84

Associations assessed by Pearson’s Chi-square and Spearman’s correlation tests

*ART* antiretroviral therapy, *PCP* primary care provider

**Table 3:** Multivariable linear regression of relationships between patient factors and models of care

	Care at nearest hospital		Care in community setting		ART from local pharmacy	
	Β coefficient (95% CI)	P value	Β coefficient (95% CI)	P value	Β coefficient (95% CI)	P value
Gender	-0.69 (-1.46 – 0.07)	0.08	-1.11 (-1.92 – -0.31)	0.007	N/A	N/A
Time of travel	-0.015 (-0.02 – -0.01)	< 0.001	N/A	N/A	N/A	N/A
Time living with HIV	-0.35 (-0.08 – 0.01)	0.10	-0.01 (-0.06 – 0.04)	0.71	-0.04 (-0.09 – 0.02)	0.19
Age	N/A	N/A	-0.94 (-1.58 – -0.29)	0.005	N/A	N/A
Born in Ireland	N/A	N/A	N/A	N/A	-0.21 (-1.14 – 0.72)	0.66
Has PCP	N/A	N/A	N/A	N/A	0.68 (-0.37 – 1.74)	0.21

Multivariable linear regression model. All variables with values shown were included in each model

### Patient Feedback on Models of Care

All participants were invited to provide unrestricted written feedback. Additional responses were provided by *n* = 206 (40%) participants. 67 of these were general feedback on their overall health and satisfaction. There were *n* = 35 responses pertaining to general care, *n* = 19 regarding communication, *n* = 6 associated with medication other than site of collection, and *n* = 79 regarding care delivery and models of care. Of the *n* = 79 concerning care delivery, *n* = 40 (51%) were supportive of the current model of care. Feedback in support of the current model included concerns regarding PCP capacity to expand their current service to PLWHIV and capability to manage the care needs of PLWHIV (*I feel there is too much for a PCP to stay abreast*,* I prefer the knowledge and experience of the [current clinic] staff; PCPs have busy schedules with sick elderly patients and I don’t think it would be fair for them to take over HIV care as well*). There were also concerns expressed regarding anonymity and confidentiality in the community *(I feel more comfortable here due to the confidentiality and respect; Pharmacists asking what medications I’m taking and why in front of a queue of people).* Stigma in the community was also a common theme (*In this clinic there is privacy*,* no stigma*,* and the staff and so lovely and accommodating; this is the only place I feel accepted*,* not stigmatised/judged*). Conversely, there were *n* = 39 in support of changing the current model to one based in the community. These included ease of access (*have more centres to make it more convenient for patients countrywide*) as well as acceptance of PCP care and medications at local pharmacy (*if HIV is controlled*,* PCP care should be good enough; it would be a step forward if PCPs could take a greater role in looking after HIV; collect medications at pharmacy*,* bloods with PCP to be sent to clinic*,* check up with clinic every couple of years if needed*).

## Discussion

This study demonstrates that alternative decentralised models of care are acceptable to specific populations of PLWHIV, in particular those who have to travel long distances and younger male patients. Providing options for healthcare delivery will promote patient-centred care, and may improve treatment adherence and reduce healthcare expenditure [15].

The median time taken to travel to and from clinic in our cohort was one hour fifty minutes. This is reflected in only 14% of respondents receiving care in their nearest hospital. Unsurprisingly, we found increased travel time to be associated with increased acceptability of receiving care in the hospital nearest to the patient. Increased travel time has been previously identified as a barrier to accessing care in PLWHIV [16]. Reduced access to care has been shown to increase when travel times are as short as thirty minutes [17]. Reduced access to care due to distance from clinics is a recognised contributor to poorer long term outcomes [18].

We demonstrate that younger age is associated with increased acceptability towards accessing HIV care in the community, as well as receiving ART from local pharmacies. Retaining younger PLWHIV in care is a recognised challenge, and accessing timely care has been identified as a barrier to care for young PLWHIV [19, 20]. Conversely, older PLWHIV often have increased healthcare needs [21, 22]. These healthcare needs have tailored interventions for PLWHIV, which often require specialist input [23, 24]. Our findings support a more accessible, agile and patient-centred model of care, with increased local access to both medical review and ART access for specific patient groups. This would likely require support from tertiary centres, which may be delivered virtually or via telehealth, with associated health infrastructure implications [25, 26]. Time living with HIV was not associated with a preference for a decentralised model of care. This may be influenced by the median age at time of HIV diagnosis, which is 39 years of age in Ireland [27]. Other factors not recorded in this study, such as disease-specific complications, severity of illness, and prior therapeutic challenges may also have influenced this. The influence of personal HIV history on attitudes towards care models should be the focus of future studies.

Male respondents were more accepting of care in the community than females. This may reflect the differing barriers accessing HIV care faced by women, in particular stigma in their local community [28, 29]. These concerns regarding stigma were also identified in our patient feedback, with the current centralised model cited as *the only place I feel accepted*,* not stigmatised/judged*. The role of stigma and differing barriers to care may also underlie our finding that being born in Ireland is associated with increased acceptability of attending a local pharmacy for ART. Migrants have well-recognised barriers to accessing care [30, 31]. The ongoing availability of centralised care, rather than fragmentation of care and medication delivery, may help overcome some of these barriers for this patient groups [32].

Our study has several limitations worth noting. We did not record any data on adherence or virologic control, and therefore cannot investigate distance travelled to clinic with treatment outcomes in our study cohort. While we captured the responses of 32% of eligible participants during the study period, our results may not be generalisable to other centres and patient populations. However, the demographics of respondents in our study are similar to that of the overall PLWHIV population in Ireland [33].

This work supports the establishment of a hybrid model of care, where patients can continue with the current standard of centralised specialist-delivered care or opt for care delivered in part or in its totality within their locality by primary care providers or internal medicine physicians. It should be used to inform future service design and improve the delivery of patient-centred care.

## Supplementary Information

Below is the link to the electronic supplementary material.

Supplementary file1

Supplementary file1
