# Supplementary material for: Suitability and Acceptability of Decentralised Care for People Living with HIV
Source: AIDS Behav. 2026 Apr 29;30(11):3624–31. doi: 10.1007/s10461-026-05144-z (PMC13727919; doi:10.1007/s10461-026-05144-z)
Supplement: Supplementary file 1 [file 10461_2026_5144_MOESM2_ESM.pdf]

Infectious Diseases Clinic

We are asking some questions about your visit to the Infectious Diseases Clinic to try and find out what you felt about the care you received.

Please be assured that whatever you say is confidential and anonymous. I would be grateful if you could hand the questionnaire to a member of staff once finished. It takes approximately 10 minutes to complete.

Thank you.

Please only complete this survey once.

Please answer questions 1-10 by ticking (√) the relevant boxes:

1. **Are you?**
   - Male ☐ 1
   - Female ☐ 2
   - Non-binary ☐ 3
   - Other ☐ 4
   - Transfemale ☐ 5
   - Transmale ☐ 6

2. **What is your Age?**
   - 15-24 years ☐ 1
   - 25-49 years ☐ 2
   - 50-65 years ☐ 3
   - 65+ years ☐ 4
   - Prefer not to answer ☐ 5

3. **What is your Region of Birth?**
   - Ireland ☐ 1
   - Sub-Saharan Africa ☐ 2
   - Latin America & Caribbean ☐ 3
   - Eastern Europe ☐ 4
   - Western Europe ☐ 5
   - Central Europe ☐ 6
   - South and Southeast Asia ☐ 7
   - Other ☐ 8 Please specify: ____________

4. **Are you currently?**
   - Employed for wages ☐ 1
   - Self-employed ☐ 2
   - Out of work and looking for work ☐ 3
   - Out of work, not looking for work ☐ 4
   - A homemaker ☐ 5
   - A student ☐ 6
   - Retired ☐ 7
   - Unable to work due to visa status ☐ 8
   - Unable to work due to health reasons ☐ 9
   - Unable to work due to other reasons ☐ 10

5. **What was your Mode of Arrival to the clinic?**

Walk	☐ 1
Car	☐ 2
Bus	☐ 3
Train	☐ 4
Taxi	☐ 5
Other	☐ 6 Please specify: ____________________

6. **Which county do you live in?**

Please specify: ____________________

7. **Approximately how long was your journey, from your door to our door at the clinic today?**

Hours __________________________ Minutes __________________________

8. **What year were you diagnosed with HIV?** _______________________________________

9. **In addition to your HIV medications, are you taking any other prescribed medications?**

Yes	☐ 1
No	☐ 2

10. **Do you know the result of your last viral load?**

Yes	☐ 1
No	☐ 2
Not sure	☐ 3

11. **Do you have a GP?**

Yes	☐ 1
No	☐ 2

12. **Do you have to pay for any part of your GP care, including bloods?**

Yes	☐ 1
No	☐ 2
Not applicable	☐ 3
Prefer not to answer	☐ 4

13. Have you shared your HIV status with any of the following? Tick all that apply

Partner	☐ 1
Family members	☐ 2
Friends	☐ 3
Dentist	☐ 4
General Practitioner	☐ 5
Prefer not to answer	☐ 6

Currently, HIV care in Ireland is delivered in a regional hospital setting.
Please read the following statements about current HIV care delivery and other potential options of delivering HIV care.

Please circle the number which best represents your opinion. Please tick (√) the not applicable box if the question does not apply to you.

14. The current location of my HIV care is convenient for me.

☹ 1 2 3 4 5 6 7 8 9 10 ☺

Disagree Agree

15. What is the name of the hospital nearest to where you live?

____

16. I would be happy to receive my HIV care at the hospital nearest to where I live.

☹ 1 2 3 4 5 6 7 8 9 10 ☺

Disagree Agree

17. I would be happy to receive my HIV care in a community healthcare setting.

☹ 1 2 3 4 5 6 7 8 9 10 ☺

Disagree Agree

18. I would be happy to receive my HIV medications from a local pharmacy.

☹ 1 2 3 4 5 6 7 8 9 10 ☺

Disagree Agree

19. I would be happy to receive all my HIV care from my GP.

☹ 1 2 3 4 5 6 7 8 9 10 ☺

Disagree Agree

Not applicable ☐

20. I would be happy to attend my GP for my HIV care at every second visit.

☹ 1 2 3 4 5 6 7 8 9 10 ☺

Disagree Agree

Not applicable ☐

21. I would be happy to receive all my HIV care from a GP of my choice.

☹ 1 2 3 4 5 6 7 8 9 10 ☺

Disagree Agree

22. I would be happy to attend a GP of my choice for my HIV care at every second visit.

☹ 1 2 3 4 5 6 7 8 9 10 ☺

Disagree Agree

23. Do you have any recommendations on how HIV care could be improved in Ireland?

END OF SURVEY

Thank you for participating in this survey
