# Supplementary material for: Suitability and Acceptability of Decentralised Care for People Living with HIV
Source: AIDS Behav. 2026 Apr 29;30(11):3624–31. doi: 10.1007/s10461-026-05144-z (PMC13727919; doi:10.1007/s10461-026-05144-z)
Supplement: Supplementary file 2 [file 10461_2026_5144_MOESM1_ESM.docx]

**Supplemental Table 1:** Acceptance of models of care by site

|  | Total (n=511) | SJH (n=379) | UHG (n=132) |  |
| --- | --- | --- | --- | --- |
| Current site is nearest hospital, yes; n (%) | 201 (39) | 128 (34) | 73 (55) | Χ^2^=21.93, p<0.001 |
| Current site is convenient; median (IQR) | 10 (7 – 10) | 10 (7 – 10) | 10 (7 – 10) | z=-0.59, p=0.55 |
| Happy to attend nearest hospital; median (IQR) | 10 (5 – 10) | 10 (4 – 10) | 10 (8 – 10) | z=-2.65, p<0.001 |
| Happy to receive care in community setting; median (IQR) | 6 (1 – 10) | 6 (1 – 10) | 7 (1 – 10) | z=-0.87, p=0.39 |
| Happy to receive medication from local pharmacy; median (IQR) | 7 (1 – 10) | 8 (1 – 10) | 5 (1 – 10) | z=2.5, p=0.01 |
| Happy to receive all care from own PCP; median (IQR) | 6 (1 – 10) | 5 (1 -10) | 8 (1 – 10) | z=-1.53, p=0.13 |
| Happy to attend PCP of choice for all visits; median (IQR) | 5 (1 – 10) | 5 (1 – 10) | 6 (1 -10) | z=-0.99, p=0.32 |

SJH=St James’s Hospital, UHG=University Hospital Galway, PCP=primary care provider

**Supplemental Table 2: Relationship between patient factors and agreeing with new model of care**

|  | Current model is convenient | | Care at nearest hospital | | Care in community setting | | ART from local pharmacy | | Care from own PCP | | Care from PCP of choice | |
| --- | --- | --- | --- | --- | --- | --- | --- | --- | --- | --- | --- | --- |
| Sex | Χ^2^=1.25 | p=0.26 | Χ^2^=7.10 | p=0.008 | Χ^2^=6.85 | p=0.009 | Χ^2^=2.10 | p=0.15 | Χ^2^=0.41 | p=0.52 | r^2^=0.0003 | p=0.99 |
| Age | r^2^=0.003 | p=0.33 | r^2^=0.01 | p=0.03 | r^2^=0.03 | p=0.002 | r^2^=0.04 | p<0.001 | r^2^=0.02 | p=0.02 | r^2^=0.02 | p=0.003 |
| Born in Ireland | Χ^2^=0.81 | p=0.37 | Χ^2^=0.58 | p=0.45 | Χ^2^=0.68 | p=0.41 | Χ^2^=10.27 | p=0.001 | Χ^2^=0.12 | p=0.73 | Χ^2^=01.23 | p=0.27 |
| Time of travel | z=7.36 | p<0.001 | z=6.87 | p<0.001 | z=1.30 | p=0.20 | z=1.14 | p=0.25 | z=-0.20 | p=0.85 | r^2^=-1.07 | p=0.28 |
| Time living with HIV | z=1.18 | p=0.24 | z=2.81 | p=0.005 | z=2.43 | p=0.02 | z=2.59 | p=0.01 | z=0.34 | p=0.73 | z=-0.12 | p=0.90 |
| Employed | r^2^=0.003 | p=0.33 | r^2^=0.001 | p=0.75 | r^2^=0.0001 | p=0.99 | r^2^=0.02 | p=0.40 | r^2^=0.003 | p=0.32 | r^2^=0.002 | p=0.43 |
| Has PCP | Χ^2^=2.80 | p=0.09 | Χ^2^=2.16 | p=0.14 | Χ^2^=2.42 | p=0.12 | Χ^2^=17.98 | p<0.001 | Χ^2^=0.71 | p=0.40 | Χ^2^=1.61 | p=0.21 |
| Disclosed status | Χ^2^=0.70 | p=0.40 | Χ^2^=1.07 | p=0.30 | Χ^2^=0.26 | p=0.61 | Χ^2^=0.001 | p=0.97 | Χ^2^=0.87 | p=0.35 | Χ^2^=2.05 | p=0.15 |

Likert responses of 8 – 10 dichotomised as “agree”. ART=antiretroviral therapy, PCP=primary care provider

**Supplemental Table 3: Multivariable logistic regression of relationships between patient factors and models of care**

|  | Care at nearest hospital | | Care in community setting | | | ART from local pharmacy | | |
| --- | --- | --- | --- | --- | --- | --- | --- | --- |
|  | Odds ratio (95% CI) | P value | Odds ratio (95% CI) | P value | Odds ratio (95% CI) | | P value |  |
| Gender | 0.60 (0.65 – 1.02) | 0.06 | 0.53 (0.32 – 0.86) | 0.01 | N/A | | N/A |  |
| Time of travel | 0.991 (0.987 – 0.995) | <0.001 | N/A | N/A | N/A | | N/A |  |
| Time living with HIV | 0.98 (0.95 – 1.01) | 0.22 | 0.99 (0.96 – 1.03) | 0.71 | N/A | | N/A |  |
| Age | 0.89 (0.59 – 1.36) | 0.60 | 0.59 (0.39 – 0.87) | 0.008 | 0.60 (0.43 – 0.84) | | 0.003 |  |
| Born in Ireland | N/A | N/A | N/A | N/A | 2.12 (1.33 – 3.38) | | 0.002 |  |

Multivariable logistic regression model. All variables with values shown were included in each model.
